# Clinical Outcomes after Oat Beta-Glucans Dietary Treatment in Gastritis Patients

**DOI:** 10.3390/nu13082791

**Published:** 2021-08-14

**Authors:** Sylwia Gudej, Rafał Filip, Joanna Harasym, Jacek Wilczak, Katarzyna Dziendzikowska, Michał Oczkowski, Małgorzata Jałosińska, Małgorzata Juszczak, Ewa Lange, Joanna Gromadzka-Ostrowska

**Affiliations:** 1 Department of Dietetics, Institute of Human Nutrition Sciences, Warsaw University of Life Sciences (SGGW-WULS), Nowoursynowska 159c, 02-776 Warsaw, Poland; katarzyna_dziendzikowska@sggw.edu.pl (K.D.); michal_oczkowski@sggw.edu.pl (M.O.); ewa_lange@sggw.edu.pl (E.L.); joanna_gromadzka_ostrowska@sggw.edu.pl (J.G.-O.); 2 Department of Gastroenterology with IBD Unit, Faculty of Medicine, University of Rzeszow, Kopisto 2A Str., 35-315 Rzeszow, Poland; r.s.filip@wp.pl; 3 Adaptive Food Systems Accelerator—Research Centre, Wroclaw University of Economics and Business, Komandorska 118/120, 53-345 Wrocław, Poland; 4 Department of Biotechnology and Food Analysis, Wroclaw University of Economics and Business, Komandorska 118/120, 53-345 Wrocław, Poland; 5 Department of Physiological Sciences, Institute of Veterinary Medicine, Warsaw University of Life Sciences (SGGW-WULS), Nowoursynowska 159, 02-776 Warsaw, Poland; jacek_wilczak@sggw.edu.pl; 6 Department of Food Gastronomy and Food Hygiene, Institute of Human Nutrition Sciences, Warsaw University of Life Sciences (SGGW-WULS), Nowoursynowska 159c, 02-776 Warsaw, Poland; malgorzata_jalosinska@sggw.edu.pl; 7 Department of Medical Biology, Institute of Rural Health, Jaczewskiego 2, 20-090 Lublin, Poland; juszczak.malgorzata@imw.lublin.pl

**Keywords:** oat beta-glucan, gastritis, inflammatory process, antioxidant properties, prebiotics, short-chain fatty acids

## Abstract

The prevalence of gastritis in humans is constantly growing and a prediction of an increase in this health problem is observed in many countries. For this reason, effective dietary therapies are sought that can alleviate the course of this disease. The objective of this study was to determine the effect of chemically pure oat beta-glucan preparations with different molar masses, low or high, used for 30 days in patients with histologically diagnosed chronic gastritis. The study enrolled 48 people of both genders of different ages recruited from 129 patients with a gastritis diagnosis. Before and after the therapy, hematological, biochemical, immunological and redox balance parameters were determined in the blood and the number of lactic acid bacteria and SCFA concentrations in the feces. Our results demonstrated a beneficial effect of oat beta-glucans with high molar mass in chronic gastritis in humans, resulting in reduced mucosal damage and healthy changes in SCFA fecal concentration and peripheral blood serum glutathione metabolism and antioxidant defense parameters. This fraction of a highly purified oat beta-glucan is safe for humans. Its action is effective after 30 days of use, which sheds new light on the nutritional treatment of chronic gastritis.

## 1. Introduction

Gastritis is usually diagnosed by the histological characteristics of tissue samples after a gastric mucosal biopsy, especially during routine upper esophagogastroduodenoscopy [1]. *Helicobacter pylori* infection is considered the main factor responsible for developing the inflammatory changes in gastric mucosa. Long-lasting gastritis predisposes to the development of gastric atrophy, intestinal metaplasia and dysplasia, which may lead to the development of the intestinal type of gastric cancer [1,2]. Apart from *H. pylori* infection, gastritis risk factors comprise inappropriate diet, non-steroid anti-inflammatory drugs (NSAIDs) and other drugs, excessive alcohol use, smoking, stress and older age [3].

There are no specific recommendations for treating chronic gastritis in the case of *H. pylori*-negative test results. Therefore, treatment regimens vary and depend on clinicians, for example, some empirically prescribe proton pump inhibitors (PPIs), some only mucosa-protecting agents, while others use both [1]. Occasionally, natural supplements or herbal medicines are also recommended to patients. On the other hand, it is still controversial for many clinicians if, for non-ulcer patients with *H. pylori* infection, eradication therapy should be advised [4]. Nevertheless, although many drugs are used to prevent and treat *H. pylori*-negative gastritis, only PPIs have proven their efficacy, especially in high-risk patients [5]. However, since PPIs are no longer considered to be completely safe for long-term use, there is still a great demand for additional treatments or/and prevention. 

Beta-glucans are polysaccharides found in the cell walls of cereals, fungi, yeast and algae. They are also considered as the soluble fraction of dietary fiber. Depending on their origin, they show differences in structure. They can create linear, branched and cyclic macroparticles that impact their biological activity. Beta-glucan present in the aleuronic layer of oat grains is a mixture of β-D-glucose unbranched chains linked by β (1-3), β (1-4) glycosidic bonds. Dissolved beta-glucans absorb large amounts of water to form high-viscosity gums [6]. They do not undergo enzymatic degradation in the stomach, creating a mucus layer, protecting against irritation and alleviating inflammation [7]. Many studies have documented their positive effect on lowering postprandial glucose and insulin levels and hypocholesterolemic results [8,9]. Beta-glucans have also been tested as a cancer treatment adjuvant [10]. Many studies have also shown that beta-glucans may be immune stimulators by activating macrophages or the stimulation of the synthesis and activation of cytokines [11,12]. Studies have also confirmed the ability of these bioactive substances to reduce infection and help to lower the death rate of surgical hospital patients due to bacterial infections or postoperative inflammatory conditions of the gastrointestinal tract [13]. However, few studies have evaluated the antioxidative and anti-inflammatory properties of beta-glucans in the case of chronic gastritis. A survey conducted with people suffering from mild chronic gastritis brings new knowledge about the safety and the possibility of using the health-promoting properties of oat beta-glucans in treating this stomach disorder.

## 2. Materials and Methods

### 2.1. Study Design 

The Research Ethics Board of the Institute of Rural Health in Lublin, Poland, approved this study (Commission Decisions No. 17/2011 and 17/2013). Informed written consent was obtained from all subjects. The study was designed as a randomized, parallel-group, double-blind, 4-week study, conducted at the Gastroenterology Outpatients’ Department and Department of GI Endoscopy of the Institute of Rural Health in Lublin (Poland). The study population was either randomly assigned to a placebo (P) group, receiving an oral dose of 100 mL 3% solution of potato starch (placebo), or to one of the treated groups receiving an oral dose of 100 mL of 3% solution of high molar mass beta-glucan (G1; 2,180,000 g/mol, purity—81.9%) or 100 mL of 3% solution of low molar mass beta-glucan (G2; 70,000 g/mol, purity—89.1%) [14]. A total of 4 clinic visits were scheduled, consisting of a screening visit (visit 1), baseline visit (visit 2), end of the treatment visit (visit 3) and follow-up visit 2 weeks after completing the treatment (visit 4). 

### 2.2. Study Subjects

The 129 participants were recruited from patients who reported to the Gastroenterology Outpatients’ Department and Department of Gastrointestinal Endoscopy of the Institute of Rural Health in Lublin to undergo an elective esophagogastroduodenoscopy (EDG) due to dyspepsia. All participants underwent a baseline screening assessment which included a medical history and physical examination. All consecutive eligible patients with an initial diagnosis of gastritis within the gastric antrum were invited to participate in the study. Finally, 48 patients of both genders (14 women and 34 men), aged 23–74 years, were recruited, randomized and subsequently treated. 

The inclusion criteria were: age between 18 and 75 years and presence of endoscopic characteristics of antral gastritis, which were then confirmed by histological examination of biopsy specimens. Subjects were excluded from the study if they met any of the following exclusion criteria: history of peptic ulcer disease; acute or erosive *gastritis*, autoimmune gastritis; prior esophagal, gastric or duodenal surgery; renal dysfunction; significant liver disease indicated by platelet count below 70,000, ascites or known gastroesophageal varices; active treatment with an H2-receptor antagonist, proton pump inhibitor, antacids or prokinetics, sucralfate; anticoagulants; fiber supplements; ingestion of aspirin or non-steroid anti-inflammatory drugs within the previous 30 days; known alcohol abuse; surreptitious drug-taking; pregnancy or lactation; the presence of alarm features, including weight loss, hematemesis, melena or rectal bleeding; significant coexisting illness or a condition that could limit the ability to participate in the study. 

The basic clinical examination was conducted and vital signs were examined at all study visits. All adverse events (AEs) were recorded at all visits. The investigators and medical experts evaluated their severity and relation to treatment and reported them to the respective authority. Participants also assessed their general well-being with a questionnaire.

### 2.3. Dietary Supplements

To obtain the fractions of 1-3, 1-4-beta-d-glucan from oat with two ranges of molar masses of 30,000–90,000 g/mol and 2,000,000–3,000,000 g/mol, a dedicated method was created. As an effect of the preliminary study, we concluded that a high molar mass fraction of 1-3, 1-4-ß-d-glucan from oat is particularly susceptible to chain length reduction due to the polymer chain’s physical degradation when subjected to mechanical stress during milling.

Briefly, to obtain a 1-3, 1-4-beta-d-glucan fraction of high molar mass, dehulled oat grains were subjected to repetitive milling (4–5 passages, with a subsequent mill gap change from 1.5 to 0.5 mm) in a corrugated roller mill. The maximum separation of the grain bran from the endosperm was obtained with minimal mechanical reduction of the bran particles. The output fraction of oat bran obtained using the milling technology was divided into two streams.

One stream was processed according to the alkaline extraction method described in a European patent [15] and led to the recovery of a 1-3, 1-4-beta-d-glucan fraction of molar mass within the range of 2,000,000–3,000,000 g/mol. The second stream was further mechanically processed for particle size reduction in the fraction. Mechanical defragmentation allows for creating controlled and repeatable processing conditions. Due to the characteristics of the oat bran layer taken off during the milling stage on the crushers (small particles of low weight, relatively flexible and not resistant), it was decided to proceed to mill it after prior freezing of the bran fraction. After 24 h at −20 °C, the fraction was subjected to intensive milling in a finger type mill (MN1 Retsch mill). The obtained fraction was frozen again (24 h at −20 °C) and subjected to another reduction by milling. A four-fold freezing and reduction operation made it possible to obtain beta 1-3, 1-4-beta-d-glucan with a fixed molar mass in the range of 30,000–90,000 g/mol by extraction by the method described in a Polish patent and described in our previous paper [16].

Molar mass was determined by an indirect method with a viscometer (Ubbelohde’s viscometer) against commercially available mass standards of oat 1-3, 1-4-beta-d-glucan (Megazyme International LTD, Wicklow, Ireland). The obtained fractions were freeze-dried (freeze-dryer LR-1 Delta 1–24 LSC), and their purity was determined by the enzymatic method using lichenase (Megazyme International LTD, Wicklow, Ireland) according to the methodology described previously [14].

The verification of the effect of administered dietary oat beta-glucan was undertaken using a 3% concentration of the pure substance in dietary supplements offered to the patients according to EFSA recommendations towards lowering blood cholesterol effect [17]. Additionally, due to the very high stickiness of beta-glucan powder after absorbing any water, it was decided to administer it in the form of a diluted gel to avoid any clogging of the esophagus during swallowing.

The gel form was prepared by dissolving the appropriate weight in 1 L of cold distilled water; then, the solution was subjected to 5 min, 750 W microwave heating to pre-dissolve beta-glucan. After microwave heating, the solution was stirred intensively with a three-leaf blender, and then hot distilled water was added and it was stirred again intensively. After reaching a homogeneous mixture, the sample was subjected to microwave radiation to adequately hydrate the beta-glucan chains for 10 min/750 W.

Then, the solution was poured into 50 mL standing test tubes (autoclavable Falcon type) and sterilized in an autoclave for 10 min at 121 °C. The two test tubes supplied 100 mL of 3% high or low molar mass beta-glucan or placebo (potato starch).

The packages given to the patients consisted of 50 mL Falcon tubes packed in boxes, closed and secured with a label marked “For testing purposes” and indicating the best-before date. Due to the lack of any preservatives that could cause additional effects in the gastric and intestinal mucosa, the shelf life was established as six weeks in preliminary tests.

For the month-long treatment of one patient, three boxes were prepared, having 20 Falcon tubes of 50 mL each. After obtaining a patient’s consent to carry out a month-long treatment, the treatments were delivered to the patients. After the end of the treatment, patients returned the packages which were intended for disposal.

Patients were instructed not to take any drugs 1 h before and 1 h after dosing. Patients were not allowed to take any fiber supplements or parapharmaceuticals during the study period. Patients were instructed to drink the delivered supplement 15–30 min before breakfast and supper. For both study groups, the diet (easily digestible diet, without beta-glucan products) was standardized during the treatment period.

### 2.4. Esophagogastroduodenoscopy and Histopathological Examination

For each subject, the same endoscopist performed the evaluations. Investigators and other personnel participating in the endoscopic assessment were blinded to the subjects’ treatment allocation. The appearance of the mucosa of the stomach and duodenum was evaluated and scored with the use of esophagogastroduodenoscopy scores (EGs) which were partially based on the Sydney grading/scoring system [18]. The initial diagnosis of chronic gastritis was categorized as superficial gastritis, erosive gastritis and atrophic gastritis according to endoscopic appearance. Two biopsies for histology were taken from the gastric antrum within 2 cm from the pylorus, one from the distal lesser curvature and the other from the distal greater curvature. One more biopsy specimen was taken for rapid urease testing (GUT Plus, Gatrex, Warsaw, Poland), confirming or excluding the presence of *H. pylori*.

Endoscopy with biopsies and histological examinations were performed before treatment (inclusion criteria) and the day after discontinuation (safety measure) of the administration. Histopathological evaluation was conducted in the ALAB Laboratory Sp. z o.o., Warsaw (Poland), and a description of each specimen was obtained.

### 2.5. Blood Sample Collection, Hematological and Biochemical Assays

Samples for biochemical analysis were collected at visits 2 and 3. After 12 h of fasting, blood samples were collected between 08:00 and 11:00 at the Department of Gastrointestinal Endoscopy of the Institute of Rural Health in Lublin. They were centrifuged, and serum (0.5 mL each portion) was kept frozen at −70 °C until the analysis.

Serum creatinine, total bilirubin concentrations, alkaline phosphatase (ALP) and gamma-glutamyltransferase (GGT) activities and basic hematological parameters were analyzed using routine laboratory techniques in the medical laboratory of Synevo Sp. z o.o. in Lublin, Poland. Plasma TNF-alpha and C-reactive protein (CRP) levels were measured using ELISA kits (Merck Millipore, Darmstadt, Germany). Plasma total antioxidant status (TAS) and the activity of glutathione peroxidase (GPx), glutathione reductase (GR) and superoxide dismutase (SOD) in blood serum were examined using Randox reagent kits (Crumlin, United Kingdom). The levels of whole blood reduced (GSH) and oxidized (GSSG) glutathione were measured according to Rebrin, Forster and Sohal [19] using a 4-channel electrochemical array for simultaneous detection. The GSH:GSSG ratio was also calculated.

### 2.6. Feces Sample Collection, Determination of the Number of Lactic Acid Bacteria (LAB) and Analysis of Short-Chain Fatty Acids (SCFAs)

Stool samples were collected in the morning after an overnight rest on the same day as the blood samples and stored at −80 °C until assayed.

Microbiological analysis was performed using a TEMPO^®^ (Biomerieux, Marcy l’Etoile, France) system to enumerate quality indicator organisms in the samples. Feces samples were homogenized in peptone water (Merck, Darmstadt, Germany) in a ratio of 1:9 using stomacher homogenizer. Then, serial dilutions of the samples were prepared, and a selective liquid culture medium for LAB was inoculated with bacteria from the homogenized feces. After incubation, the bacterial suspension was placed in TEMPO^®^ enumeration cards containing 48 wells across three different dilutions to automatically determine the most probable number (MPN) of lactic acid bacteria. Filled cards were incubated aerobically at 37 °C for 48 h.

Analysis of short-chain fatty acids in feces was performed using HPLC with UV detection. The mobile phase for the isocratic elution of SCFAs was 15 mM monobasic sodium phosphate:methanol (80:20) on Hypersil BDS 150 × 4.6 mm, 5 µm column (Sigma-Aldrich (St. Louis, MO, USA)) at a mobile phase flow rate of 1.2 mL/min and detection at 224 nm. The quantitative and qualitative analyses of propionic, butyric, acetic, formic and lactic acid were performed using pure chemical standard SCFAs (Sigma-Aldrich (St. Louis, MO, USA)).

### 2.7. Statistical Analysis

The data were analyzed using Statistica 13.3 PL (TIBCO Software Inc., Tulsa, OK, USA). Normal distribution and equality of variances were tested for in all the data. Data that presented non-normal distribution were transformed with a common logarithm. A two-way repeated measures ANOVA was used for comparisons between groups (placebo, beta-glucan with high molar mass, beta-glucan with low molar mass) and the two time points (before and after dietary intervention) of the experiment (group × time point 3 × 2; with repeated measures on time points). Fisher’s test was used for post hoc comparisons. Statistical significance was accepted for *p* < 0.05. The obtained results are presented as mean ± standard error of the mean (SEM).

Fisher’s linear discriminant analysis (F-LDA) using R statistical software v. 3.3.3. (www.rproject.org/ (accessed on 23 May 2021)) (R: The R Project for Statistical Computing) analyzing the interaction between investigated parameters at the two time points was also performed.

## 3. Results

### 3.1. Study Population

A total of 129 patients were screened, of which 48 started in the study. The included patients consisted of women and men aged 23–74 with BMI 17.0–38.8 kg/m^2^, who were randomly assigned to three following dietary groups, with 16 patients each: placebo group (P), oat beta-glucan with a high molar mass group (G1) and oat beta-glucan with low molar mass (G2). Dietary intervention with 3% of an aqueous suspension of potato starch (P group) or oat beta-glucan with high molar mass (G1 group) or oat beta-glucan with low molar mass (G2 group) was recommended for 30 days. Compliance with the treatment regimen was calculated from the number of study products dispensed to the subjects, and the number of study products returned to the study site. No difference in compliance was observed.

The age and anthropometric parameters of the study population are shown in Table 1. None of the examined parameters differed between the groups, between men and women or before vs. after dietary intervention.

### 3.2. Well-Being and Adverse Effects during the Study

There was no significant difference in subjective assessment of the well-being of patients during the study. There were no statistically significant differences in well-being between the dietary groups. From 75% (placebo group) to 78% (G1 group) and 79% (G2 group) of patients declared good and very good well-being, none of the patients declared bad or very bad well-being. The overall incidence of adverse events (AEs) was similar across the study groups. None of the AEs led to the discontinuation of the study and no serious adverse event (SAE) was recorded. The most commonly occurring AEs during the treatment period were dyspepsia (seven in G2, six in G1 and five in the placebo group), upper abdominal pain or discomfort (eight in G2, four in G1 and ten in the placebo group), upper pulmonary tract infections (one in the G1 group and one in the placebo group) and an increase in arterial pressure (two in the G1 group and two in the placebo group). Both dyspepsia and upper abdominal pain and discomfort were usually mild, and the symptoms disappeared within a few days. Therefore, dyspepsia did not generally lead to discontinuation of the study. The increase in arterial blood pressure was mild and occurred episodically in subjects with a history of arterial hypertension. No clinically relevant treatment-related events were recorded during the study.

### 3.3. Histological Examination of Biopsy Specimens

In each case, the following features were microscopically evaluated using the visual analogue scales (according to the updated Sydney System): inflammation (presence of lymphocytes and plasma cells within the lamina propria) and its activity (presence of neutrophils), glandular atrophy, intestinal metaplasia (complete or incomplete) and presence of *Helicobacter pylori* in the superficial mucus [9]. Evaluated featured were scored in the range 0–3 (0, none; 1 mild; 2, moderate; 3, severe).

Before the dietary intervention, all patients participating in the study had histologically diagnosed chronic gastritis. The changes found in the gastric mucosa include lymphocytes infiltration within the lamina propria (score 1–2), the presence of neutrophils (score 1–2) and the presence of *Helicobacter pylori* (80% of patients were negative). After 30 days of beta-glucans, or placebo administration only in the G1 group, a reduction in inflammation manifested by a decrease in the infiltration of lymphocytes (score 0–1) and neutrophils (score 0) was found.

### 3.4. Peripheral Blood Hematology and Biochemical Parameters

No statistical differences (ANOVA) related to the type of dietary intervention or duration of treatment were found in the hematological parameters, except platelet count with a higher value in group G2 (Table 2), as well as the chosen biochemical blood serum parameters (Table 3).

### 3.5. Blood Serum Immunological Parameters

**Figure 1:**
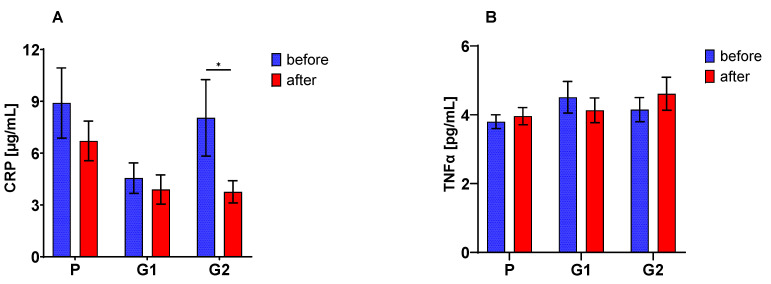
Serum C-reactive protein (CRP) (**A**) and TNFα (**B**) concentration (mean ± SE); The asterisk (*) denotes a statistically significant difference after vs. before dietary intervention, set at *p* < 0.05.

A decreased concentration of CRP in the peripheral blood serum of all patients after 30 days of nutritional intervention was found. These changes were confirmed by the significant ANOVA analysis (*p* < 0.02), but only in the G2 group were these changes statistically significant (Figure 1A). Neither the type of supplement nor the duration of nutritional intervention (ANOVA NS) had a considerable effect on TNF-alpha concentration (Figure 1B).

### 3.6. Peripheral Blood Redox Balance Parameters

**Figure 2:**
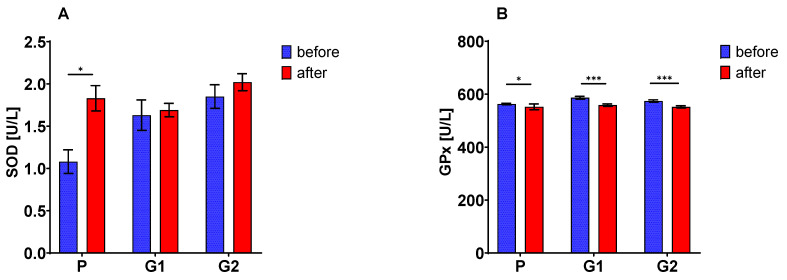

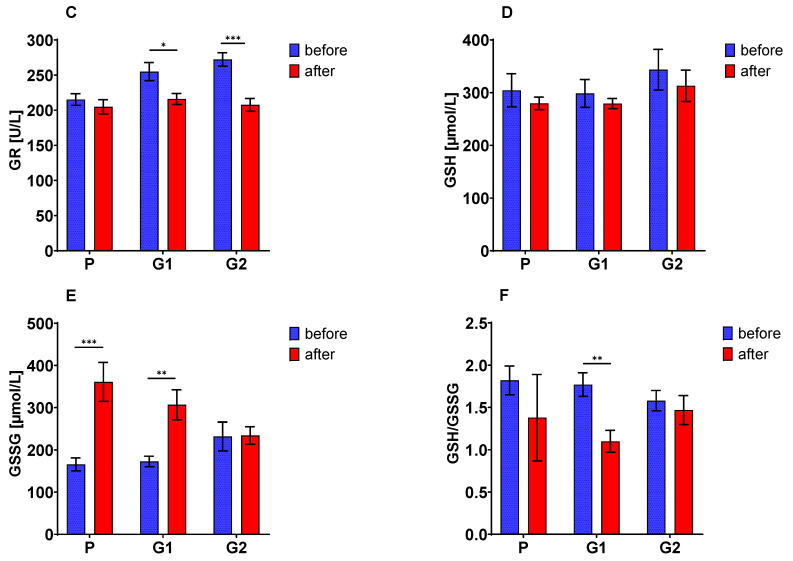
Blood superoxide dismutase (SOD) (**A**), glutathione peroxidase (GPx) (**B**) and reductase (GR) (**C**) activities; serum reduced (GSH) (**D**) and oxidized (GSSG) (**E**) glutathione and GSH to GSSG ratio (**F**). The asterisks (*, **, ***) denote a statistically significant difference after vs. before dietary intervention, set at *p* < 0.05; *p* < 0.01; *p* < 0.001, respectively.

The activity of SOD (Figure 2A) after a 30-day nutritional intervention increased (ANOVA, *p* = 0.0000), with significant changes only in the group of patients with placebo treatment (*p* < 0.002). The activity of both GPx (Figure 2B) and GR (Figure 2C) after 30 days of nutritional intervention significantly decreased (ANOVA, in both cases *p* < 0.0001), regardless of the molar mass of the supplement used (in the case of GPx) or only when oat beta-glucan preparations were used (in the case of GR, there was a significant effect of the supplement type *p* < 0.04).

The concentration of the reduced form of glutathione (Figure 2D) did not change as a result of the 30-day nutritional intervention (ANOVA NS). In contrast, the concentration of the oxidized form of this compound (Figure 2E) increased significantly in patients from the placebo and G1 groups (ANOVA, *p* = 0.0000; interaction of type of supplement vs. duration of use *p* < 0.03). In the G1 group the GSH/GSSG ratio (Figure 2F) decreased, which confirmed the significance of the analysis of variance (ANOVA, *p* = 0.0000).

### 3.7. The Fecal Number of LAB and SCFA Concentration

**Figure 3:**
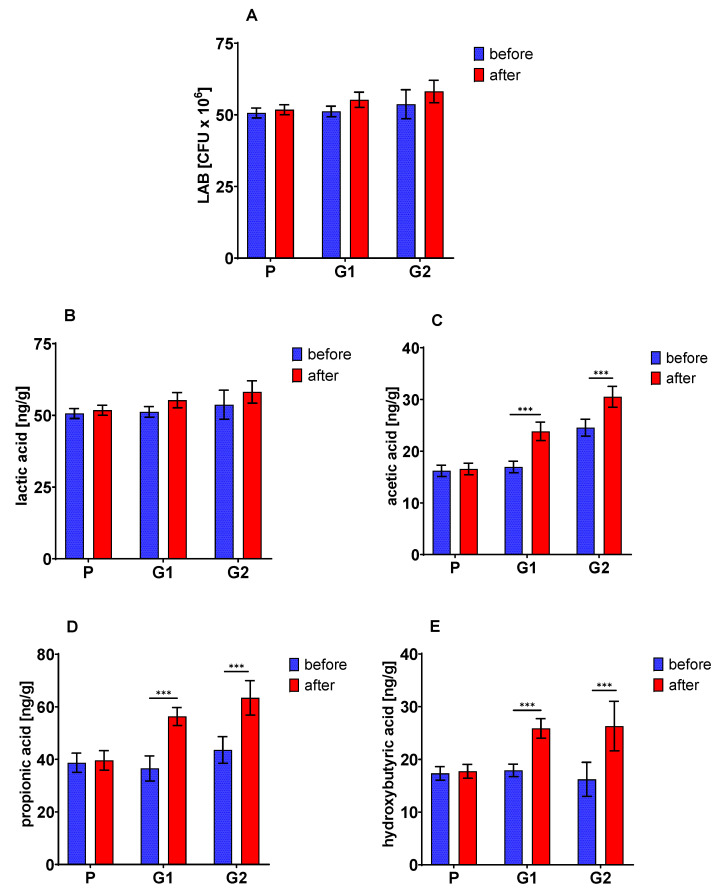
Lactic acid bacteria (**A**) and short-chain fatty acids (lactic, acetic, propionic and hydroxybutyric) (**B**–**E**) in feces. The asterisks (***) denote a statistically significant difference after vs. before dietary intervention, set at *p* < 0.001.

The number of lactic acid bacteria increased after 30 days of oat beta-glucan with high molar mass consumption, but it was not a statistically significant change. The analysis of variance also did not confirm any substantial changes in this parameter (Figure 3A). Additionally, no significant differences were found due to the 30-day nutritional intervention in the fecal concentration of lactic acid (Figure 3B), while the concentration of acetic acid (Figure 3C), propionic acid (Figure 3D) and hydroxybutyric acid (Figure 3E) increased significantly in patients consuming both oat beta-glucan fractions (ANOVA, in all cases *p* = 0.0000).

### 3.8. Fisher’s Linear Discriminant Analysis (FLD)

**Figure 4:**
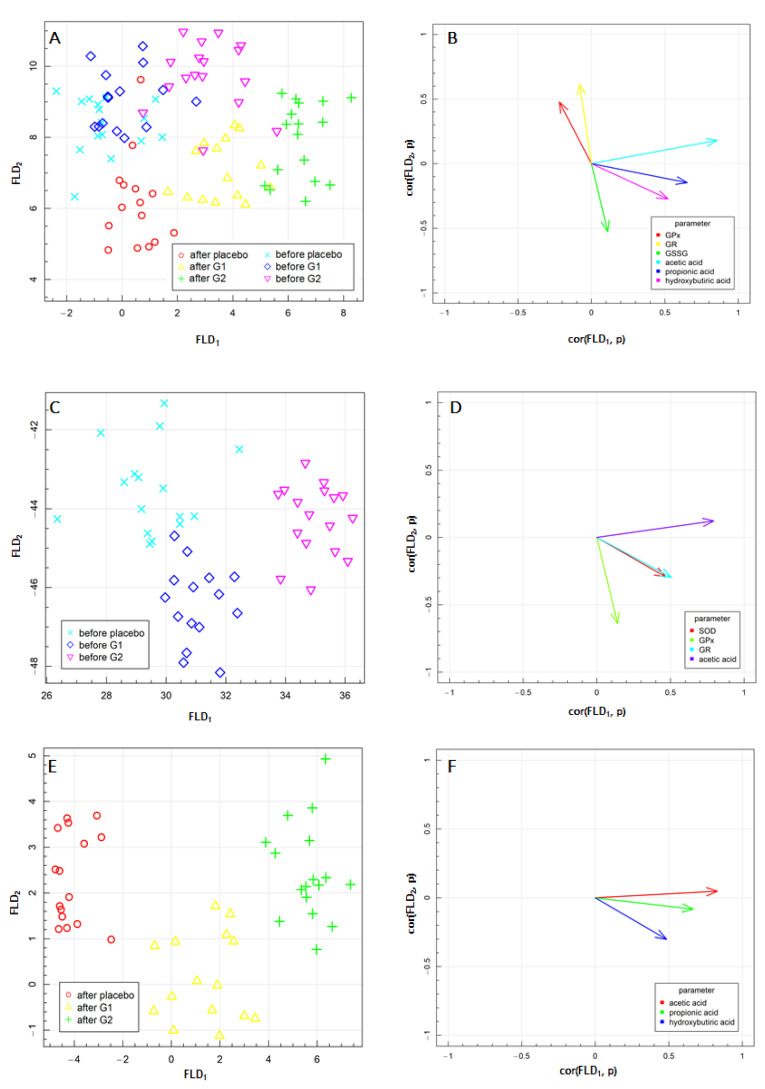
Fisher’s Linear Discriminant (FLD) Analysis: (**A**,**C**,**E**) experimental data on the plane spanned by two the most data separating FLDs and (**B**,**D**,**F**) parameters contributing the most to FLDs. (**A**,**B**)—before and after the beta-glucans intervention; (**C**,**D**)—before the beta-glucans intervention; (**E**,**F**)—after 30 days of the beta-glucans intervention.

Fisher’s linear discriminant analysis (FLD) was performed to explore other differences in the above parameters associated with beta-glucan supplementation. The results of FLD obtained for the experimental data are presented in Figure 4. FLD was used to optimize the linear combination of antioxidative defense and oxidative stress parameters and SCFA level to obtain the best separation of the experimental groups, supplemented with G1 or G2 oat beta-glucan or placebo at the beginning of the treatment and after one month of the supplementation. The FLD test provides the most efficient linear combination of analyzed parameters needed to separate groups treated with both fractions of oat beta-glucan. The results of the linear discriminant analysis at two time points are shown in Figure 4A,C. In each figure, three treatment groups are separated. The data are shown between the linear combinations of parameters (FLDs), marked as FLD1 and FLD2, which separate the best predefined group.

The results of the FLD together and at two experimental time points are shown in Figure 4A,C,E. In Figure 4A, six experimental groups are isolated, and in Figure 4C,D, three experimental groups per time point are separated. The data are presented in the space between the linear combinations of parameters (FLDs), marked as FLD1 and FLD2, ensuring the optimal separation of the predefined groups. Figure 4B,D,F show the correlation vectors of the studied parameters with FLD1 and FLD2 predictors, which establish the direction and influence of a given parameter on the separation of the experimental groups: for all aggregated data (Figure 4A,B), before treatment with both fractions of oat beta-glucans or placebo (Figure 4C,D) and after one month of supplementation (Figure 4E,F). The graphs highlight the parameters that proved to have the most critical impact on the separation of data. The biological marker compatible with a particular vector caused the data to move in the direction indicated by the vector. The FLD complemented and confirmed the statistical analysis of ANOVA, which showed the increased concentration of acetic acid, propionic acid and hydroxybutyric acid after 30 days of high molar mass oat beta-glucan consumption.

FLD performed for all data obtained in the present study (Figure 4A,B) confirmed that the factors that most differentiated these experimental groups were SCFAs, in particular acetic and propionic acid, as well as antioxidative defense enzymes including GPx and GR activity in the blood, which was also confirmed by ANOVA results.

The FLD provided information about combining the above parameters, which are helpful to distinguish the groups of data acquired before and after oat beta-glucan supplementation with G1 or G2 beta-glucans in the vertical plane (FLD2). Additionally, as indicated in the horizontal plane (FLD1), they did not enable the separation of the placebo group from the other groups before dietary intervention (Figure 4A) but simultaneously showed that it is possible after one month of nutritional enrichment with oat beta-glucan. Therefore, we decided to perform separate FLDs for each time point of the present study.

At the beginning of the research, the separation of the groups was possible, but the correlation vector power is low. The horizontal plane (FLD1) allowed the separation of the placebo group from the group that took low molecular mass oat beta-glucan (G2), as well as the separation of the low molecular mass beta-glucan group and the group that was supplemented with high molar mass beta-glucans (G1). Blood serum activity of GPx presented the most significant influence on FLD2, while acetic acid had a strong effect on FLD2. Both FLDs were impacted by the blood serum activity of SOD and GR. Based on this, it can be concluded that at the beginning of the experiment, all the groups were characterized by similar propionic and hydroxybutyric acid levels.

After one month of dietary intervention (Figure 4E,F), the analyzed parameters differentiated the experimental groups much more than at the earlier time point. Interestingly, all markers that allowed us to separate the experimental groups belong to carboxylic acids. The placebo group, as well as both groups that consumed oat beta-glucans, were separated by FLD1 and FLD2. The acetic and propionic acid levels in the stool significantly impacted FLD1, whereas hydroxybutyric acid influenced FLD2 (Figure 4F).

## 4. Discussion

Our study is the first randomized, double-blind experiment to evaluate the effects of oat beta-glucans in chronic gastritis in humans. The obtained results showed that the 30-day dietary supplementation with beta-glucans of different molar masses in a dose of 3 g/day resulted in molar mass-dependent changes in some immunological and redox balance parameters in peripheral blood serum and SCFA concentration in the stool of patients with gastritis. These supplements did not cause any significant changes in the hematological and biochemical blood indices and only a slight normalization of histopathological changes in patients from the G1 group. It should be emphasized that the dietary supplementation of beta-glucans in patients with gastritis did not adversely affect their well-being and did not exacerbate the clinical gastrointestinal symptoms, which proves the harmlessness of these highly purified beta-glucan preparations isolated with the use of alkaline extraction from oat grains.

This inflammation is driven by genetic factors, bacterial pathogenicity, patient age or nutritional factors, among others [20]. Maintaining the integrity of the gastric mucosa also depends on exposure to reactive oxygen species, which can increase lipid peroxidation and cause damage to this mucosal barrier [21,22,23]. For the endoscopic and histopathological assessment of chronic gastritis, the Sydney System is commonly used, which allows for a standardized approach to biopsy interpretation and allows for a clinically correct diagnosis [24]. Using the Sydney System, chronic gastritis was confirmed in all patients enrolled in the experiment, which was only slightly reduced in those who used oat beta-glucan with a high molar mass as a dietary supplement for 30 days.

Chronic gastritis often does not manifest changes in hematological and biochemical parameters. However, as shown in a few studies, gastritis caused by an increased presence of *Helicobacter pylori* causes changes in the percentage of platelets and the average platelet volume. However, due to the large population variability of these parameters caused by the gender and age of patients, they currently do not play an essential role in carrying out the diagnosis of gastritis [25]. Any chronic inflammation activates platelets, and the mean platelet volume (MPV) reflects this effect well. Still, there are more and more studies showing that MPV and platelet counts do not differ between *H. pylori*-positive and *H. pylori*-negative patients. There were also no differences or correlations between MPV and the severity of gastritis according to the Sydney classification [26]. As was shown in pediatric gastritis, no significant differences in hematological parameters, including platelet count and mean volume, were found between control and stomach inflammation patients, except severe gastritis, which manifested with a decrease in hemoglobin and hematocrit levels [27]. The lack of changes in the hematological and biochemical blood picture found in our patients confirms, on the one hand, mild or moderate gastritis and, on the other hand, good tolerability and safety of the use of both oat beta-glucan preparations. There was also no change in the patients’ general well-being during oat beta-glucan supplementation compared to the placebo group, which indicated that these cereal polysaccharides are well tolerated in patients suffering from inflammation of the gastric mucosa.

Maintaining adequate antioxidant protection in stomach cells is possible by antioxidant enzyme activities, such as superoxide dismutase (SOD), peroxidase (GPx) and glutathione reductase (GR). Additionally, glutathione, a non-enzymatic antioxidant that should dominate in its reduced form, is crucial [28]. Glutathione is an endogenous component that plays a crucial role in critical physiological processes, helping to protect against redox balance, reducing oxidative stress, enhancing metabolic detoxification and regulating immune function. The latter aspect of glutathione metabolism is especially essential in the context of inflammatory diseases of individual components of the digestive system. Much evidence indicates that glutathione metabolism is a biomarker and indicator of the effectiveness of nutritional management in gastrointestinal disorders [29,30]. Our study focused on the GSH/GSSG parameter calculated based on the quantitative analysis of glutathione in the reduced (GSH) and oxidized (GSSG) forms and the activity of two critical enzymes in glutathione metabolism: glutathione reductase and glutathione peroxidase. Selenocysteine-containing glutathione peroxidases use glutathione to reduce H_2_O_2_ or lipid peroxides, generating oxidized glutathione [31].

In turn, regeneration of GSH from GSSG is catalyzed by glutathione reductase (GR) in the GSH redox cycle. The reduction of GSSG occurs at the expense of NADPH, produced by the pentose phosphate pathway from glucose oxidation [32,33]. Our study confirmed that in patients with diagnosed gastritis, the use of beta-glucans for 30 days, regardless of their molar mass, caused changes in glutathione metabolism: blood serum activity of both GPx and GR decreased, and in the case of high molar mass beta-glucan, the GSSG blood serum concentration was increased and the quotient GSH/GSSG ratio was decreased. As the levels of GSH decrease in states with generated oxidative stress, this would suggest increased oxidative stress after beta-glucan diet treatment; however, other parameters of oxidative stress (i.e., SOD, TAS) do not confirm this. In our opinion, the endogenous mechanism of glutathione synthesis is disturbed due to the decrease in the pool of available substrates for glutathione synthesis. Inflammation of the gastrointestinal tract causes a reduction in dietary availability, including amino acids. The primary substrates for the synthesis of glutathione are glutamate, cysteine and glycine, which are used in cell inflammation for high-energy processes. The reduced concentration of substrates for the synthesis of glutathione is responsible for oxidative stress and dietary supplementation, e.g., glutamine and cysteine reduce the effects of oxidative stress by increasing the parameter of the antioxidant potential of the blood [34]. This is confirmed by the fact that glutathione reductase activity is reduced, leading to a decreased amount of GSH from GSSG. As noted in some studies [35,36], pathological conditions such as inflammation and neoplasms lower GSH levels (and thus raise the GSSG/GSH ratio) of glutathione metabolism and it seems to be more folded up than expected. For example, the sustained constant activity of GPx does not guarantee a continuous concentration of both GSH and GSSG [37].

Our previous studies have confirmed the beneficial effect of oat beta-glucan in different gastrointestinal diseases in animal models [38]. In our previous study on rats with experimentally DSS-induced gastritis, we also found a beneficial effect of oat beta-glucan consumption which confirmed the important molar mass-dependent role of these cereal polysaccharides in maintaining the antioxidative defense of stomach tissue undergoing inflammation [39]. Consumption of low molar mass oat beta-glucan decreased SOD activity and concentration of GSH in the stomach tissue of animals with induced gastritis. In contrast, consumption of a high molar mass fraction caused a lowering of the concentration of GSSG but did not affect the concentration of GSH in the group of rats with gastritis. In rats, all parameters were analyzed in the stomach tissue, while in patients they were measured in peripheral blood, which could have resulted in slightly different results. It should be added that in studies on animal models of gastritis, we used the same oat beta-glucans as in patients suffering from this gastric inflammation. Both high and low molar mass beta-glucans were integral rat feed additives, while gastritis patients were given these polysaccharides in the form of a drinking gel. Different forms of beta-glucan preparations consumed by humans and animals could also have contributed to the differences in the obtained results.

The differences in the obtained results also concern the immunological parameters. In rats with gastritis, high beta-glucan consumption resulted in a significant reduction in the concentration of TNF-alpha in the stomach tissue [39], while in humans, the concentration of this immune parameter in the blood did not change.

In the stool of patients diagnosed with gastritis and consuming oat beta-glucans, the number of lactic acid bacteria (LAB) and the level of intestinal microbiota metabolites—short-chain fatty acids (SCFAs)—were tested. Although the subject of the study was not the colon itself, which is to a greater extent a beneficiary of fecal LAB changes, there are reasons to believe that any method influencing the fecal LAB profile has positive effects on the whole organism [40,41,42]. It is true that we measured only the LAB number without identification and assignment to families of intestinal bacteria. Still, this observation confirmed that LAB changes in the stool are an individual feature, dependent on many factors. Statistical analysis of the LAB results showed no statistically significant differences between the study groups. Still, the exact individual changes between the beginning and end of beta-glucan treatment showed changes within individual patients. These changes are mainly individual differences in the consumption of these food products, which are the primary substrate for LAB biodiversity in the stool. This variability has covered up the effect of beta-glucans. A too-small group of patients did not allow us to show significant differences in our entire study. Individual changes in the number of LAB are consistent with changes in metabolites of the whole intestinal microflora.

In our studies, the concentrations of SCFAs in the stool, acetic, hydroxybutyric and propionic acids, are statistically significantly correlated with both the pathological state and the beta-glucan diet therapy. The lactic acid concentration in stool remained constant, regardless of the type of beta-glucans used. Changes in the concentration of individual SCFAs and their mutual proportions are a characteristic feature of quantitative changes in microbiota populations, especially in pathophysiological states. Still, it seems impossible to clearly state which SCFAs play the most critical role in the pathomechanism of these changes at the present stage. The results of our studies to date clearly indicate that beta-glucans in the model of LPS-induced inflammation cause changes in SCFA profiles in the feces of rats [43]. The direction of these changes is similar in the case of the described use of beta-glucans. Likewise, we observed an increase in propionic, lactic and hydroxybutyric acid concentrations. Their increased presence in the stool determines their increased availability for enterocytes and demonstrates a positive effect on their metabolism.

On the one hand, evidence points to the fact that fecal propionic acid levels are affected by irritable bowel syndrome (IBS) [44]. Still, propionic acid is of great interest due to its substantial immunomodulatory effects. Its supplementation reduces the exacerbation of colitis in murine models, suggesting that its modulation may be a therapeutic intervention in inflammatory bowel disease [45]. On the other hand, studies by Ormsby and co-workers highlight the potential risk of widespread use of propionic acid as an antimicrobial, demonstrating this mechanism through the increased virulence and persistence of the pathotype adherent-invasive *E. coli* (AIEC) strain in a mouse colitis model [46]. The results of our studies indicate only the increased concentrations of SCFAs in the stool as a result of beta-glucan treatment. They are not the basis for concluding their safety.

Our study had several strengths. First, it was conducted in a double-blind, randomized trial. Second, we used highly purified oat beta-glucan with specific molar mass ranges. The duration of the study, 30 days, was sufficient to assess the safety of the systemic use of beta-glucans in patients with gastritis, as well as the therapeutic effect of these cereal polysaccharides. The limitations of our study concern primarily a heterogeneous group in terms of age and gender. Another downside is the lack of biochemical analyses in the stomach tissue, where antioxidative and anti-inflammatory defense processes occurring in chronic inflammation of the gastric mucosa are primarily found.

## 5. Conclusions

Our study demonstrated a beneficial effect of oat beta-glucans with high molar mass in chronic gastritis in humans, resulting in reduced mucosal damage and healthy changes in SCFA fecal concentrations and peripheral blood serum glutathione metabolism and antioxidant defense parameters as well. This fraction of a highly purified oat beta-glucan is safe for the body. Its action is effective after 30 days of use, which sheds new light on the nutritional treatment of chronic gastritis.

## Figures and Tables

**Table 1:** Age and anthropometric parameters of study population (mean ± SEM).

Parameters	Group	Mean ± SEM	Min	Max
Age (years)	Placebo	50.75 ± 3.61	23.00	68.00
	G1	46.00 ± 3.82	23.00	74.00
	G2	50.41 ± 3.24	27.00	68.00
Height [cm]	Placebo	170.88 ± 2.19	156.00	183.00
	G1	169.93 ± 2.10	152.00	178.00
	G2	171.41 ± 2.19	156.00	184.00
Weight [kg]	Placebo	86.63 ± 2.37	73.20	109.60
	G1	79.93 ± 3.95	54.00	116.50
	G2	79.17 ± 3.56	64.90	113.10
BMI [kg/m^2^]	Placebo	29.82 ± 1.00	23.43	38.83
	G1	27.72 ± 1.26	17.04	36.80
	G2	26.88 ± 0.93	20.97	36.51

**Table 2:** Hematological parameters of study population (mean ± SEM).

Parameters	Group	Before Treatment	After Treatment
WBC × 10^3^/µL	Placebo	6.22 ± 0.34	5.81 ± 0.27
	G1	6.36 ± 0.45	6.25 ± 0.47
	G2	5.90 ± 0.20	6.20 ± 0.30
RBC × 10^6^/µL	Placebo	4.94 ± 0.05	4.91 ± 0.05
	G1	5.03 ± 0.10	4.92 ± 0.07
	G2	4.86 ± 0.08	4.91 ± 0.08
Hemoglobin [g/dL]	Placebo	14.51 ± 0.38	14.48 ± 0.27
	G1	15.25 ± 0.29	14.97 ± 0.29
	G2	14.71 ± 0.27	14.86 ± 0.30
Hematocrit [%]	Placebo	42.84 ± 0.87	42.69 ± 0.52
	G1	44.18 ± 0.68	43.37 ± 0.77
	G2	42.60 ± 0.72	43.08 ± 0.73
Lymphocytes × 10^3^/µL	Placebo	1.97 ± 0.14	1.93 ± 0.13
	G1	1.95 ± 0.12	1.93 ± 0.11
	G2	1.83 ± 0.10	2.08 ± 0.09
Platelets × 10^3^/µL	Placebo	204.19 ± 9.5	199.94 ± 8.07
	G1	213.23 ± 16.7	227.57 ± 19.0
	G2	236.65 ± 13.2 *	250.24 ± 13.6 *

* Statistically significant differences *p* < 0.05.

**Table 3:** Blood serum biochemical parameters of study population (mean ± SEM).

Parameters	Group	Before Treatment	After Treatment
Creatinine [mg/dL]	Placebo	0.74 ± 0.02	0.78 ± 0.03
	G1	0.81 ± 0.03	0.83 ± 0.04
	G2	0.76 ± 0.04	0.78 ± 0.04
Bilirubin [mg/dL]	Placebo	0.64 ± 0.08	0.65 ± 0.05
	G1	0.71 ± 0.12	0.64 ± 0.09
	G2	0.65 ± 0.09	0.80 ± 0.13
ALT [U/L]	Placebo	33.30 ± 4.50	32.68 ± 6.70
	G1	24.77 ± 3.00	23.24 ± 3.10
	G2	26.82 ± 1.90	26.59 ± 2.19
AST [U/L]	Placebo	25.63 ± 1,48	26.37 ± 4.00
	G1	28.48 ± 4.69	26.14 ± 3.90
	G2	22.47 ± 1.12	22.82 ± 1.24

## Data Availability

The data that support the findings of this study are available on request from the corresponding author (S.G.).
